# SOCIAL COGNITION AND AWARENESS IN ALZHEIMER’S DISEASE

**DOI:** 10.1093/geroni/igz038.441

**Published:** 2019-11-08

**Authors:** Marcia Dourado, Tatiana Belfort, José Simões Neto

**Affiliations:** 1 Institute of Psychiatry - Universidade Federal do Rio de Janeiro, Rio de Janeiro, Brazil; 2 Sociology and Political Science Department, Federal University of Santa Catarina,, Florianópolis, Santa Catarina, Brazil

## Abstract

Social cognition is the capacity to interpret and predict another’s behavior according to beliefs, intentions, and emotions, and the ability to decode environmental stimuli in order to be better able to adapt to new situations. A key question is the relationship between social cognition and awareness in dementia. This study aimed to investigate the relation between social and emotional functioning (SEF) and awareness in Alzheimer's disease (AD). In a cross-sectional design, a consecutive series of 50 people with mild to moderate AD and their 50 family caregivers were assessed. The study variables were awareness, SEF, neuropsychiatric symptoms, cognition, working memory, quality of life, functional activities, presence of depressive symptoms, and caregivers’ burden and cognition. We found a significant difference between self-rated SEF and informant-rated SEF. In 56% of the cases, self-rated SEF was lower than the informant-rated SEF. People with AD mostly (56%) had mildly impaired awareness of disease, 20% had moderate impaired awareness of disease, and 6% were unaware of the disease. A multivariate linear regression examined the association between informant-rated SEF score and the variables. The social functioning and relationship domain of awareness and informant-rated QoL of people with AD were significantly associated with informant-rated SEF. Conclusion: The relationship between informant-rated SEF and awareness of social functioning and relationship supports the multidimensional nature of awareness. SEF and awareness of social functioning shows that they are comprised of judgments related to perceptions about oneself and values qualitatively different from awareness of memory or functionality, which can be directly observed.

